# Consequences of ignoring clustering in linear regression

**DOI:** 10.1186/s12874-021-01333-7

**Published:** 2021-07-07

**Authors:** Georgia Ntani, Hazel Inskip, Clive Osmond, David Coggon

**Affiliations:** 1 Medical Research Council Lifecourse Epidemiology Unit, University of Southampton, Southampton, UK; 2 Medical Research Council Versus Arthritis Centre for Musculoskeletal Health and Work, Medical Research Council Lifecourse Epidemiology Unit, University of Southampton, Southampton, UK; 3 NIHR Southampton Biomedical Research Centre, University of Southampton and University Hospital Southampton NHS Foundation Trust, Southampton, UK

**Keywords:** Clustering, Linear regression, Random intercept model, Consequences, Simulation, Comparison, Bias

## Abstract

**Background:**

Clustering of observations is a common phenomenon in epidemiological and clinical research. Previous studies have highlighted the importance of using multilevel analysis to account for such clustering, but in practice, methods ignoring clustering are often employed. We used simulated data to explore the circumstances in which failure to account for clustering in linear regression could lead to importantly erroneous conclusions.

**Methods:**

We simulated data following the random-intercept model specification under different scenarios of clustering of a continuous outcome and a single continuous or binary explanatory variable. We fitted random-intercept (RI) and ordinary least squares (OLS) models and compared effect estimates with the “true” value that had been used in simulation. We also assessed the relative precision of effect estimates, and explored the extent to which coverage by 95% confidence intervals and Type I error rates were appropriate.

**Results:**

We found that effect estimates from both types of regression model were on average unbiased. However, deviations from the “true” value were greater when the outcome variable was more clustered. For a continuous explanatory variable, they tended also to be greater for the OLS than the RI model, and when the explanatory variable was less clustered. The precision of effect estimates from the OLS model was overestimated when the explanatory variable varied more between than within clusters, and was somewhat underestimated when the explanatory variable was less clustered. The cluster-unadjusted model gave poor coverage rates by 95% confidence intervals and high Type I error rates when the explanatory variable was continuous. With a binary explanatory variable, coverage rates by 95% confidence intervals and Type I error rates deviated from nominal values when the outcome variable was more clustered, but the direction of the deviation varied according to the overall prevalence of the explanatory variable, and the extent to which it was clustered.

**Conclusions:**

In this study we identified circumstances in which application of an OLS regression model to clustered data is more likely to mislead statistical inference. The potential for error is greatest when the explanatory variable is continuous, and the outcome variable more clustered (intraclass correlation coefficient is ≥ 0.01).

**Supplementary Information:**

The online version contains supplementary material available at 10.1186/s12874-021-01333-7.

## Introduction

Clinical and epidemiological research often uses some form of regression analysis to explore the relationship of an outcome variable to one or more explanatory variables. In many cases, the study design is such that participants can be grouped into discrete, non-overlapping subsets (clusters), such that the outcome and/or explanatory variables vary less within clusters than in the dataset as a whole. This might occur, for example, in cluster-randomised controlled trials (with the units of randomisation defining clusters), or in a multi-centre observational study (the participants from each centre constituting a cluster). The extent to which a variable is “clustered” can be quantified by the intra-class correlation coefficient (ICC), which is defined as the ratio of its variance between clusters to its total variance (both between and within clusters) [1].

**Fig. 1:**
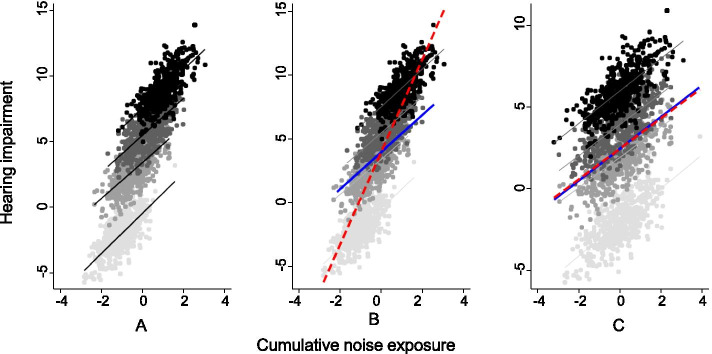
Two hypothetical relationships of hearing impairment to cumulative noise exposure in four cities. Units for noise exposure and hearing impairment have been specified arbitrarily for ease of presentation. Data for each city are distinguished by the shading of data points. **A** and **B** Depict the same hypothetical dataset. In **A**, only cluster-specific regression lines are indicated, while in **B** summary regression lines have been added for the full dataset a) when clustering is ignored (dotted red line), and b) after adjustment for clustering (solid blue line). **C** Shows a second dataset in which the relationship of hearing impairment to cumulative noise exposure in each city is as in **A** and **B**, but distribution of noise exposures is the same in each city. Again, the dotted red line represents the summary regression line when clustering is ignored, and the solid blue line, that after adjustment for clustering

Clustering has implications for statistical inference from regression analysis if the outcome variable is clustered after the effects of all measured explanatory variables are taken into account. If allowance is not made for such clustering as part of the analysis, parameter estimates and/or their precision may be biased. This possibility can be demonstrated by a hypothetical study of hearing impairment and noise exposure, in which observations are made in four different cities (clusters), as illustrated in Fig. 1. In this example, the effect of cumulative noise exposure on hearing impairment is the same within each city (i.e. the regression coefficient for hearing impairment on noise exposure is the same in each cluster), but the distribution of the exposure differs across cities (Fig. 1A). After allowance for noise exposure, hearing impairment differs by city, such that it varies more between the clusters than within them. An analysis that ignored this clustering would give a misleading estimate for the regression coefficient of hearing loss on noise exposure (Fig. 1B with cluster-unadjusted and cluster-adjusted effect estimates superimposed on Fig. 1A). Moreover, even if the distribution of noise exposures in each city were similar, so that the regression coefficient was unbiased, its precision (the inverse of its variance) would be underestimated, since variance would be inflated by failure to allow for the differences between clusters (at the intercept) (Fig. 1C).

Where, as in the example above, the number of clusters is small relative to the total number of participants in the study sample, a categorical variable that distinguishes clusters can be treated as an additional explanatory variable in the regression model [2]. However, when the number of clusters is larger (again relative to the total number of participants), use of the cluster variable as an additional explanatory variable in the regression model can seriously reduce the precision with which effects are estimated (because more degrees of freedom are used). In such circumstances, an alternative approach is to assume that cluster effects are randomly distributed with a mean and variance that can be estimated from the data in the study sample. Random intercept models assume that the effects of explanatory variables are the same across all clusters, but that the intercepts of regression lines differ with a mean and variance which can be estimated from the study data, along with the effect estimates of primary interest. Random slope models assume that the effects of explanatory variables also differ between clusters, with a mean and variance that can be estimated.

In recognition of the potential implications of clustering for statistical inference, there has been a growth over recent years in the use of statistical techniques that allow for clustering [3–5]. Nevertheless, many studies still ignore clustering of observations [6–10]. Recent systematic reviews have reported that clustering was taken into account in only 21.5% of multicentre trials [11] and 47% of cluster randomised trials [12]. This may in part reflect computational challenges and statistical complexities [13], but, perhaps because of a lack of clarity about the effects of ignoring clustering, authors have omitted to discuss the limitations of their chosen analytical techniques.

Several studies have investigated implications of ignoring clustering in statistical inference, most being based on analysis of real data [14–21]. To date, no study has systematically investigated the extent to which bias can occur in effect estimates when clustering is ignored, the determinants of that bias, or the exact consequences for the precision of estimates according to different distributions of the explanatory variable and, in particular, the extent to which the explanatory variable varies within as compared with between clusters. Such variation can be more nuanced in observational studies (in which researchers have less control over the distribution of explanatory variables), than in clinical trials where the main explanatory variable either varies only between clusters (as in cluster randomised trials), or exhibits minimal variation between as compared with within clusters (as when individual randomisation produces balanced prevalence of the explanatory variable across clusters).

The first aim of the research described in this paper was to assess in detail the implications for effect estimates (regression coefficients), and their precision (characterised by standard errors (SEs)), when a linear regression analysis exploring the relation of a continuous outcome variable to an explanatory variable fails to account for clustering. The second aim was to describe coverage by 95% confidence intervals and rates of Type I error in the same setting. These research questions were explored through simulation studies, which were designed to cover a range of scenarios that might occur in observational research, including variable degrees of clustering in the explanatory variable.

## Methods

In the simplest case, in which there is a single explanatory variable, the ordinary least squares (OLS) linear regression is specified by a model of the form:

1 $${y}_{i}={\beta }_{0}+{\beta }_{1}{x}_{i}+{e}_{i}$$

For a continuous outcome and a single explanatory variable, the random intercept (RI) multi-level model can be viewed as an extension of the OLS model, and is specified as:

2 $$\begin{array}{l}{y}_{ij}={\beta }_{0j}+{\beta }_{1}{x}_{ij}+{e}_{ij}\\ ={\beta }_{0}+{\beta }_{1}{x}_{ij}+{e}_{ij}+{u}_{j}\end{array}$$

where the index $i$ refers to the individual and the index $j$ to the cluster, and ${\beta }_{0j}={\beta }_{0}+{u}_{j}$, the estimate of the intercept for cluster $j$. The term ${u}_{j}$ represents the error for cluster $j$ around the fixed intercept value of ${\beta }_{0}$, and is assumed to be normally distributed with ${u}_{j}|{x}_{ij}\sim N\left(0,{SD}_{u}^{2}\right)$. The term ${e}_{ij}$ represents the additional error within the cluster, also referred to as the individual level error term, with ${e}_{ij}|{x}_{ij},{u}_{j}\sim N\left(0,{SD}_{e}^{2}\right)$.

As described in the introduction, ICC is a measure which characterises the extent to which the outcome variable ${y}_{ij}$ is similar within clusters, given the distribution of the explanatory variable ${x}_{ij}$ [4]. For a continuous outcome variable, and with the nomenclature used above, the ICC is defined as $\mathrm{I}\mathrm{C}\mathrm{C}=\frac{{SD}_{u}^{2}}{{SD}_{u}^{2}+{SD}_{e}^{2}}$ [1].

To explore the study questions, simulated datasets were generated according to the assumptions of the RI model (as specified in Eq. 2). For each simulation, both the number of clusters and the number of observations per cluster were set to 100. For simplicity, the size of the effect of ${x}_{ij}$ on ${y}_{ij}$ was arbitrarily set to 1 (${\beta }_{1}=1)$, and the average value of ${y}_{ij}$ when ${x}_{ij}$= 0 was arbitrarily set to 0 (${\beta }_{0}=0)$.

Separate simulations were generated for a continuous and a binary explanatory variable ${x}_{ij}$. To set values for a continuous explanatory variable, ${x}_{ij}$, in a cluster $j$, an individual level variable generated as ${x}_{0ij}\sim N\left(0,1\right)$ was added to a cluster-specific variable generated as ${shift}_{j}\sim N\left(0, {{SD}_{shift}}^{2}\right)$, so that ${x}_{ij}={x}_{0ij}+{shift}_{j}$. A total of 1,000 values for ${SD}_{shift}$, derived as $\sim U\left[\mathrm{0,20}\right]$, were each used to generate 100 simulated samples, giving a total of 100,000 samples.

For a binary explanatory variable ${x}_{ij}$, we set the prevalence in each of the 100 clusters within a sample to be the sum of a constant “target prevalence” (the same in all clusters) and a cluster-specific variable ${shift}_{j}\sim N\left(0, {{SD}_{shift}}^{2}\right)$. In this case, 500 values of ${SD}_{shift}$, derived as $\sim U\left[\mathrm{0,0.05}\right]$, were each used to generate cluster prevalence rates for 100 simulated samples, giving a total of 50,000 samples for each of four values for target prevalence (0.05, 0.1, 0.2 and 0.4). Where a negative value was generated for a cluster prevalence, it was set to zero. Values for ${x}_{ij}$ within a cluster were then set to achieve the designated prevalence for that cluster (with rounding as necessary). For example, if the prevalence assigned to a cluster was 0.223, then the first 22 values for ${x}_{ij}$ in the cluster were set to 1, and the remaining 78 to 0.

For both continuous and binary ${x}_{ij}$, corresponding values for the outcome variable ${y}_{ij}$ were generated according to Eq. 2. For this purpose, the individual-level error terms were drawn from a random standard normal distribution $\left(N\left(\mathrm{0,1}\right)\right)$, and the cluster-level error terms were drawn from a random normal distribution with mean zero and variance ${SD}_{{u}_{j}}^{2}$. We aimed to explore outcomes according to the degree of clustering within study samples, and to this end, we specified six target ranges for sample ICC (0.0005–0.00149, 0.0025–0.00349, 0.005–0.0149, 0.025–0.0349, 0.05–0.149 and 0.25–0.349). Simulated data were therefore generated for six different values for ${SD}_{{u}_{j}}$ (0.0316, 0.05485, 0.1005, 0.1759, 0.3333 and 0.6547) chosen to give expected values for the sample $ICC$ at the mid-points of the target ranges (0.001, 0.003, 0.01, 0.03, 0.1 and 0.3 respectively). Throughout the remainder of this report, the six target ranges are labelled by these midpoint values. Where, by chance, the ICC for a sample fell outside its target range, the sample was discarded, and replaced by a new sample generated using the same value for $s{d}_{shift}$. This process continued until the ICC fell inside the target range.

Further details of the algorithms used to generate simulated samples are presented in supplementary files (Appendices A and B for continuous and binary explanatory variable respectively).

For each simulated sample, two linear regression models were fitted; an OLS model which ignored the clustering (Eq. 1), and a RI multi-level model which allowed for clustering effects (Eq. 2). For each of the models, the regression coefficient and its standard error (SE) were estimated. To assess bias in effect estimates from the two models, we calculated differences between regression coefficients estimated by the two methods (${\beta }_{1}^{RI}$ and ${\beta }_{1}^{OLS}$) and the “true” value of 1 (i.e. the value used in the algorithm to generate simulated samples), as has been done previously [22]. To explore how deviations from the “true” value were affected by the clustering of the explanatory variable, they were plotted against the dispersion (expressed as standard deviation (SD)) of the cluster mean values of continuous ${x}_{ij}$ (${\stackrel{-}{x}}_{j}$) across the clusters of each sample, and against the dispersion (again expressed as SD) of cluster prevalence rates of binary ${x}_{ij}$ across the clusters of each sample. For both continuous and binary ${x}_{ij}$, lower dispersion indicated less variation of the explanatory variable across clusters and therefore lower clustering of the explanatory variable (since within-cluster variance of ${x}_{ij}$ remained constant). In addition, descriptive statistics were produced for the distributions of deviations from the “true” value across samples, according to ICC (ICC referring to the pattern of variation in the outcome variable after allowance for the explanatory variable), and for a binary explanatory variable, also according to the target prevalence of ${x}_{ij}$.

To compare the precision of effect estimates derived from the two models, the ratios of their SEs (${SE}^{RI}/{SE}^{OLS}$) were calculated. Again we explored how findings varied according to ICC, clustering of the explanatory variable, and, where the explanatory variable was binary, its target prevalence.

The coverage of the 95% confidence intervals for the regression coefficient ${\beta }_{1}$ from the two methods was assessed by calculating the percentage of the estimated confidence intervals that included the” true” value that had been used in the simulations. A method was considered to have appropriate coverage if 95% of the 95% confidence intervals included the “true” value of the effect ${\beta }_{1}$ (i.e. 1). Deviations from nominal coverage could reflect bias in estimates of effect, unsatisfactory standard errors [23], or both.

To assess impacts on Type I error, simulations were repeated using the same numbers of simulated samples (i.e. 100,000 simulations for each ICC target range for continuous ${x}_{ij}$, and 50,000 simulations for each target prevalence and ICC target range for binary ${x}_{ij}$), this time assuming no association between ${x}_{ij}$ and ${y}_{ij}$ (i.e. we set ${\beta }_{1}=0$).The percentage of datasets for which the null hypothesis was rejected at a 5% significance level in OLS and RI modelling were compared according to ICC.

All simulations and analyses were conducted using Stata software v12.1.

## Results

### Bias in regression coefficients

**Fig. 2:**
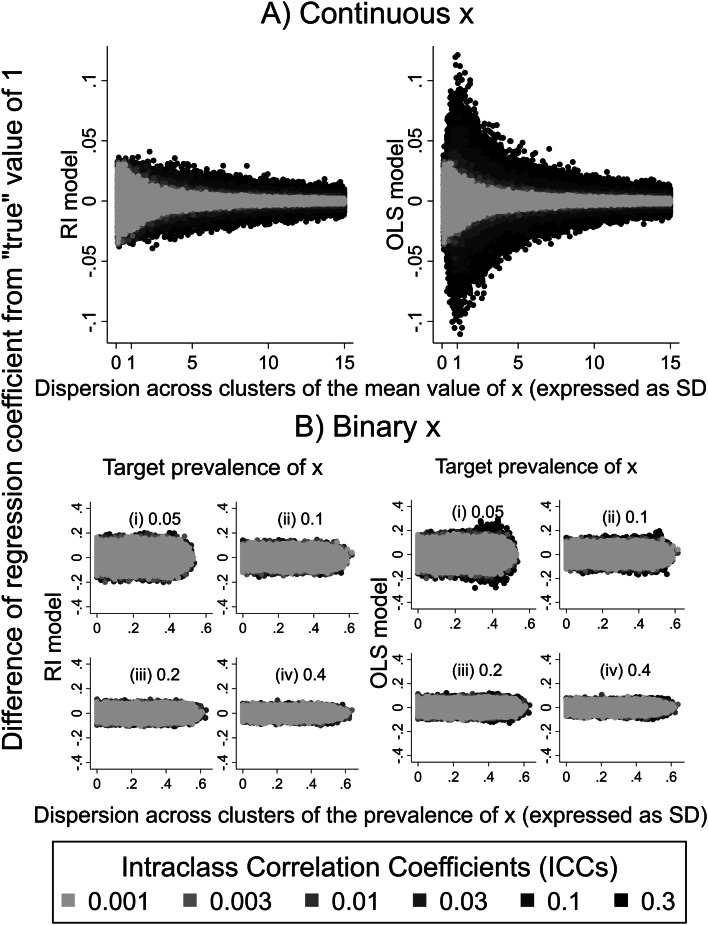
Difference from “true” value of 1 of regression coefficients estimated from RI and OLS models (${\beta }_{1}^{RI}$ and ${\beta }_{1}^{OLS}$) plotted against dispersion (expressed as SD) of the mean value/prevalence of ${x}_{ij}$ across the clusters within each sample. Results for different levels of intraclass correlation coefficient are distinguished by shades of grey as indicated in the legend. **A** Continuous ${x}_{ij}$. **B** Binary ${x}_{ij}$

Figure 2 illustrates how regression coefficients estimated from the two linear models differed from the “true” value of 1. The two subplots of the figure (A and B) correspond to the two types of explanatory variable (continuous and binary respectively), and the different shades of grey represent different ICC levels with darker shades corresponding to simulated results for higher ICCs.

In all cases, differences from the “true” value of 1 were on average zero, indicating that both models produced unbiased estimates of the regression coefficient. However, with a continuous explanatory variable, divergence from the “true” value tended to be greater for the OLS than for the RI model, especially for higher ICC and for lower dispersion of the mean value of ${x}_{ij}$ across the clusters within a sample (Supplementary Table 1). With a binary explanatory variable, divergence from the nominal value was again greatest for high ICCs (see also Supplementary Table 2), but there was no strong relationship to dispersion of the mean prevalence of ${x}_{ij}$ across clusters, and average divergence differed less between the two models.

### Ratio of standard errors

**Fig. 3:**
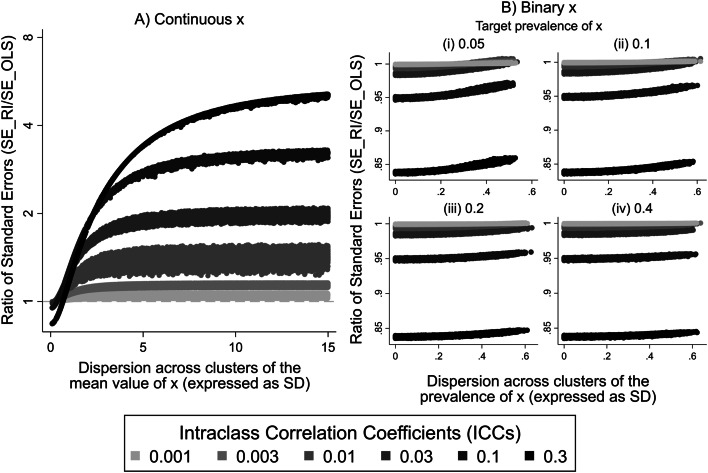
Ratios of standard errors estimated from RI and OLS models (${\mathrm{S}\mathrm{E}}_{{\mathrm{\beta }}_{1}^{\mathrm{R}\mathrm{I}}}$/${\mathrm{S}\mathrm{E}}_{{\mathrm{\beta }}_{1}^{\mathrm{O}\mathrm{L}\mathrm{S}}}$) plotted against dispersion (expressed as SD) of the mean value/prevalence of ${x}_{ij}$ across the clusters within each sample. Results for different levels of intraclass correlation coefficient are distinguished by shades of grey as indicated in the legend. **A** Continuous ${x}_{ij}$. **B** Binary ${x}_{ij}$

The ratios of SEs derived from the RI and OLS models (${SE}_{{\beta }_{1}^{RI}}/{SE}_{{\beta }_{1}^{OLS}}$) were examined in relation to the dispersion of the mean value/prevalence of the continuous/binary explanatory variable ${x}_{ij}$ across the clusters within the sample, and are presented in Fig. 3. As in Fig. 2, levels of ICC are represented by different shades of grey, with lighter shades corresponding to lower ICCs and darker shades to higher ICCs. Subplots A and B illustrate the ratios of SEs when ${x}_{ij}$ was continuous and binary, respectively.

For a continuous variable ${x}_{ij}$, the ratio took its minimum value for the smallest dispersion of cluster mean values of ${x}_{ij}$ (${\stackrel{-}{x}}_{j}$) and increased towards a plateau as that dispersion increased. The minimum and maximum values of the ratio of the SEs (the latter corresponding to the plateau value) were ICC-dependent, higher ICCs resulting in lower minimum and higher maximum values for the ratio. The dispersion of ${\stackrel{-}{x}}_{j}$ at which the ratio of SEs approached its plateau was also ICC-dependent, being higher for larger ICCs. For very small values of dispersion of ${\stackrel{-}{x}}_{j}$, the minimum value of the ratio of the SEs was approximately one for small levels of ICC and was less than one for higher ICCs. Particularly for small values of the dispersion of ${\stackrel{-}{x}}_{j}$ and ICC $\cong$ 0.10 or 0.30, the ratio of SEs was < 1, meaning that SEs from RI models were smaller than from OLS models.

When ${x}_{ij}$ was binary, the ratios of the SEs were below one for most of the situations examined, indicating that the SEs of the regression coefficients estimated from the RI model were smaller than those from the OLS model in most circumstances. The ratio of the SEs achieved its minimum value for the smallest dispersion of the prevalence of ${x}_{ij}$ across the clusters within a sample, and increased progressively as that dispersion increased. For small ICCs (< 0.1), the SEs from the two models were very similar. However, as ICC increased to 0.1 or higher the ratio of the SEs decreased to values much lower than 1. For constant ICC, comparison of subplots of Fig. 3B, shows that the rate of increase of the ratio of the SEs was higher for lower target prevalence rates of the ${x}_{ij}$.

### Coverage of 95% confidence intervals

Table 1 shows the extent to which 95% confidence intervals covered the “true” effect of a continuous explanatory variable on the outcome (${\beta }_{1}$=1), when derived from the two statistical models. Results are presented separately for different levels of ICC, and for fifths of the distribution of the dispersion of cluster means of the explanatory variable across the clusters of the sample.

**Table 1:** Coverage (%) of “true” effect ${\beta }_{1}$ = 1 by 95% confidence intervals derived from the RI and OLS models according to fifths of the distribution of dispersion (expressed as SD) of the cluster means of a continuous ${x}_{ij}$ within samples

	**Lowest fifth of distribution**		**2**^**nd**^** fifth of distribution**		**3**^**rd**^** fifth of distribution**		**4**^**th**^** fifth of distribution**		**Highest fifth of distribution**		**Total**	
	**RI**	**OLS**	**RI**	**OLS**	**RI**	**OLS**	**RI**	**OLS**	**RI**	**OLS**	**RI**	**OLS**
**0.001**	95.04	94.37	95.00	94.09	95.33	94.10	95.15	93.99	94.92	93.84	95.08	94.08
**0.003**	95.14	93.14	95.37	92.33	95.20	91.83	95.53	92.25	95.42	91.99	95.33	92.30
**0.01**	94.99	88.47	94.64	83.95	94.75	83.65	94.74	83.72	94.90	84.07	94.80	84.75
**0.03**	94.59	76.21	95.11	68.79	94.80	67.62	95.06	67.57	94.74	67.15	94.87	69.39
**0.1**	94.68	59.58	94.80	45.45	94.86	44.83	94.39	44.73	95.04	44.37	94.76	47.80
**0.3**	94.84	41.32	94.53	28.06	94.95	28.24	94.64	26.98	94.79	27.41	94.75	30.36

Irrespective of ICC and type of explanatory variable, coverage with the RI model was approximately 95% (for continuous ${\mathrm{x}}_{\mathrm{i}\mathrm{j}}$: range across ICC levels 94.75–95.33%; for binary ${\mathrm{x}}_{\mathrm{i}\mathrm{j}}$: range across ICC levels and prevalence rates of ${\mathrm{x}}_{\mathrm{i}\mathrm{j}}$ 94.72–95.15%). For a continuous ${x}_{ij}$, coverage for the OLS model was close to 95% for very low ICC but decreased with increasing levels of ICC. For the highest ICC level examined (ICC = 0.3), OLS gave a notably poor coverage of 30%. For a given ICC, coverage of 95% confidence intervals did not vary much according to dispersion of the mean value of ${x}_{ij}$ across clusters, although it was somewhat higher in the bottom fifth as compared with the rest of the distribution of dispersions.

**Fig. 4:**
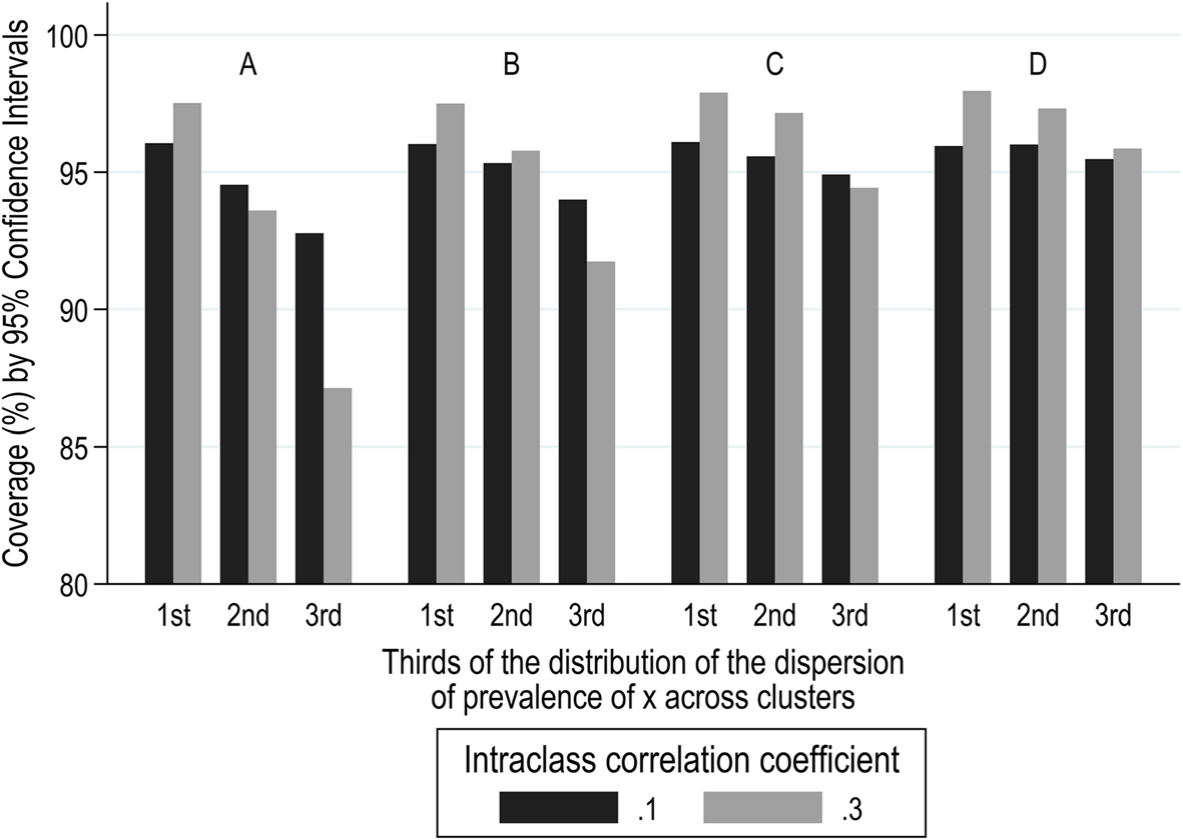
Coverage (%) by 95% confidence intervals from the OLS model for ICC = 0.1 and 0.3, according to target prevalence of $x$ (A) 0.05, B) 0.10, C) 0.20, and D) 0.40), and thirds of the distribution of the dispersion (expressed as SD) of the prevalence of ${x}_{ij}$ across the clusters within each sample

For a binary ${x}_{ij}$, coverage from the OLS model was close to 95% (range 94.7 to 95.2%) for ICC ≤ 0.03. However, as ICC increased, coverage from the OLS model deviated from the nominal value of 95%. As shown in Fig. 4, when ICC was 0.1 or 0.3, coverage was on average lower for lower target prevalence of ${x}_{ij}$; it fell below the nominal value of 95% for 0.05 target prevalence of ${x}_{ij}$ and it increased to values higher than 95% for 0.40 target prevalence of ${x}_{ij}$ (comparison of the four sub-plots of the figure). Also, for any given target prevalence of ${x}_{ij}$, coverage was lower for increasing dispersion of prevalence of ${x}_{ij}$ across clusters. Variation of the average coverage by target prevalence of ${x}_{ij}$ and dispersion of prevalence of ${x}_{ij}$ across clusters was higher when ICC was higher (ICC = 0.3) than when it was lower (ICC = 0.1). The smallest and the largest values of coverage were 87 and 98% and they were observed when the target prevalence of ${x}_{ij}$ was 0.05, ICC = 0.3, and in the lowest and highest thirds respectively of the distribution of dispersion of prevalence of ${x}_{ij}$ across clusters. Coverage as high as 98% was also seen in the lowest third of the distribution of dispersion of prevalence of ${x}_{ij}$ across clusters for the other prevalence rates (0.10, 0.20, and 0.40) explored when ICC was high (ICC = 0.3).

### Type I error

To assess the frequency of Type I error, defined as incorrect rejection of a true null hypothesis, under the OLS and the RI multi-level models, simulations were repeated assuming no association between the explanatory variable ${x}_{ij}$ and the outcome variable ${y}_{ij}$ (${\beta }_{1}=0$).

**Fig. 5:**
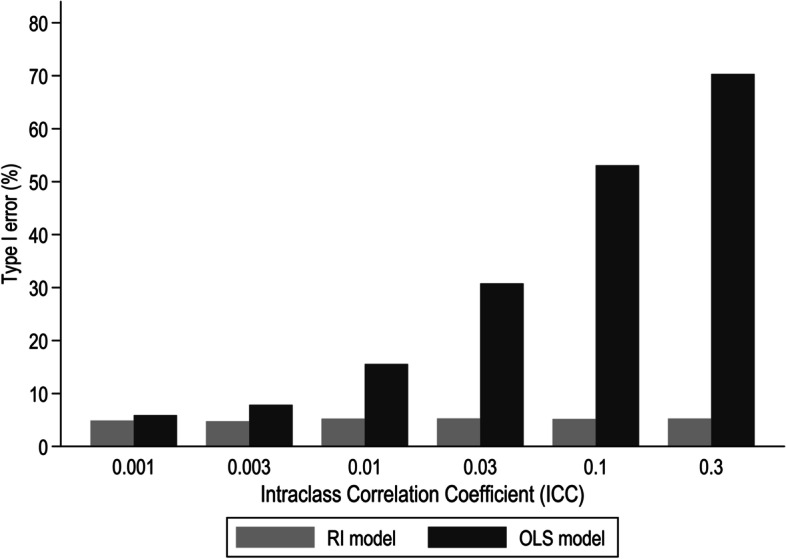
Percentage (%) of simulated samples for which the null hypothesis was rejected according to level of ICC when ${x}_{ij}$ was continuous and no association was assumed between outcome and explanatory variable

Figure 5 shows the percentage of simulated samples for which the null hypothesis was rejected at a 5% significance level for varying levels of ICC, when ${x}_{ij}$ was continuous. Using the RI multi-level model, the association between ${x}_{ij}$ and ${y}_{ij}$ was statistically significant in approximately 5% of the datasets for all ICCs. However, with the OLS models, Type I error rose with ICC. For a very small ICC, Type I error was close to the nominal value of 5%, but it increased rapidly as the ICC increased, reaching $\sim 70\%$ for ICC $\cong$ 0.30. Type I error did not vary much by dispersion of the cluster mean values of ${x}_{ij}$ within samples, but was lowest in the lowest fifth of the distribution of dispersion (Supplementary Table 3).

**Fig. 6:**
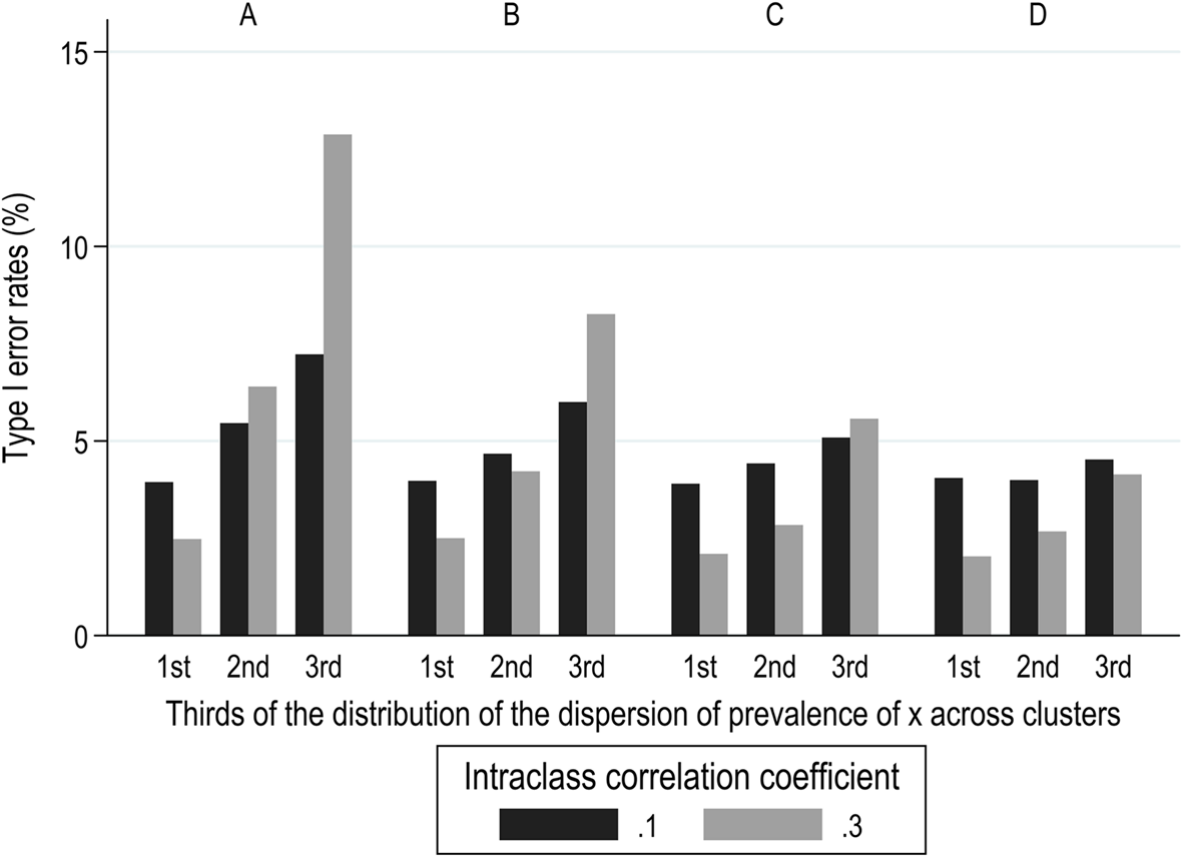
Type I error rates (%) from the OLS model for ICC = 0.1 and 0.3, by target prevalence rates of ${x}_{ij}$ (**A** 0.05, **B** 0.10, **C** 0.20, and **D** 0.40), and thirds of the distribution of the dispersion (expressed as SD) of prevalence of ${x}_{ij}$ across the clusters within each sample

When the explanatory variable ${x}_{ij}$ was binary, Type I error rates from the OLS model varied very little around the nominal level of 5% when ICC values were less than 0.1; the average value was 5% and varied from 4.8 to 5.3% for different ICC values (< 0.1), target prevalence rates of ${x}_{ij}$, and dispersion of prevalence of ${x}_{ij}$ across clusters. However, for ICC values of 0.1 and 0.3, Type I error rates diverged from 5%. This is illustrated in Fig. 6 for the four target prevalence rates of ${x}_{ij}$ (subplots A, B, C, and D of the figure), and for thirds of the distribution of dispersion of prevalence of ${x}_{ij}$ across clusters. For small dispersion of prevalence rates of ${x}_{ij}$ (bottom third of the distribution), Type I error was lower than 5%, and it increased as dispersion increased. This trend was more prominent for lower values of target prevalence of ${x}_{ij}$, and for ICC = 0.3 compared to ICC = 0.1. The smallest and the largest values of Type I error were 2 and 13% and they were observed when the prevalence of ${x}_{ij}$ was 0.05 and in the lowest and highest thirds respectively of the distribution of dispersion of prevalence of ${x}_{ij}$ across clusters.

## Discussion

In this study we focused on the implications of ignoring clustering in statistical inference regarding the relationship between a continuous outcome and a single explanatory variable ${x}_{ij}$. For each of two types of ${x}_{ij}$ (continuous and binary), we fitted RI and OLS models and explored: the deviation of effect estimates from the “true” value that was used in generating the samples; their relative precision; the extent to which their 95% confidence intervals covered the “true” value; and the frequency of Type I error when simulations assumed that there was no association between the outcome and explanatory variable. Our interest was principally in implications for analysis of data from observational studies, and we specified the generation of simulated samples to encompass a range of scenarios that might be encountered in real observational data. In particular, we considered varying degrees of clustering not only in the outcome variable (measured by ICC), but also in the explanatory variable. The latter was quantified in terms of the SD across clusters of the mean or prevalence of the explanatory variable within each cluster (its variance within clusters being fixed).

With both continuous and binary ${x}_{ij}$, where the “true” effect was non-zero, we found that irrespective of ICC, both RI and cluster-unadjusted OLS models on average gave estimates of effect close to the “true” value (i.e. they were unbiased). However, deviations from the “true” value were greater for higher ICC. For continuous ${x}_{ij}$, they tended also to be greater for the OLS than the RI model, and when the explanatory variable was less clustered. However, with a binary explanatory variable, deviations from the “true” value showed no strong relationship to the level of clustering of the explanatory variable, and average divergence from the “true value" differed less between the two models.

SEs for effect estimates from cluster-unadjusted OLS differed from those derived from RI models, the differences being driven mainly by ICC levels and the extent to which the explanatory variable was clustered. For higher clustering of the explanatory variable, the SEs of regression coefficients from the RI model were generally larger than from the cluster-unadjusted OLS model. When ${x}_{ij}$ was continuous, the ratio of SEs (${SE}_{{\beta }_{1}^{RI}}/{SE}_{{\beta }_{1}^{OLS}}$) was highest (> 4) for a high ICC (0.3). However, the apparently greater precision of OLS method was not universal. For low clustering of the explanatory variable, OLS regression gave larger SEs than RI modelling, particularly for higher ICCs (> 0.03). With both continuous and binary ${x}_{ij}$, SEs from RI modelling were more than 15% lower than those from OLS regression for the highest ICC value (ICC = 0.3) and the lowest clustering of the explanatory variable.

The rates of coverage of 95% confidence intervals for estimates of effect, whether of a continuous or a binary ${x}_{ij}$, when derived from a RI model were at the nominal level of 95%, irrespective of other parameters. When ${x}_{ij}$ was binary, the cluster-unadjusted OLS model also resulted in an appropriate coverage of 95% confidence intervals provided ICC was low ($\le 0.01$). However, for higher values of ICC, coverage varied around the nominal value of 95% (range: 87–98%) depending on the overall prevalence and the dispersion of the cluster-specific prevalence rates of ${x}_{ij}$. In contrast, when ${x}_{ij}$ was continuous, the model that failed to account for clustering resulted in much poorer coverage rates, especially as ICC increased, and they were as low as 30% for ICC = 0.3.

Setting the effect of ${x}_{ij}$ on the outcome variable to zero allowed exploration of the frequency of Type I error. With the RI model, Type I error was close to 5% in all of the scenarios explored. When ${x}_{ij}$ was continuous, we found that failure to allow for clustering increased rates of Type I error, and that the inflation of Type I error was particularly pronounced (up to 70%) when the degree of clustering was high (ICC = 0.3). In contrast, when ${x}_{ij}$ was binary, Type I error under the OLS model was close to the expected value of 5% for low ICC (< 0.1). However, when ICC was high (0.1 or 0.3), Type I error rates varied more widely around 5%, with values as low as 2% (for low target prevalence of ${x}_{ij}$ and small dispersion of its prevalence across clusters) and as high as 13% (for low target prevalence of ${x}_{ij}$ and large dispersion of its prevalence across clusters).

The analysis for each specification of parameters (expected ICC, dispersion of mean or prevalence of ${x}_{ij}$ across clusters, and (for binary ${x}_{ij}$) overall prevalence of ${x}_{ij}$) was based on a large number of simulated samples (100,000 for each of six target ranges of ICC for continuous ${x}_{ij}$, and 50,000 for each of 24 combinations of target ICC and target prevalence of ${x}_{ij}$ for binary ${x}_{ij}$), each of which comprised 10,000 observations grouped in 100 equally sized clusters. By using such a large sample size (larger than in many epidemiological investigations), we reduced random sampling variation, making it easier to characterise any systematic differences between the two methods of analysis. However, the approach may have led to underestimation of the maximum errors in effect estimates that could arise from OLS as compared with multi-level modelling. Moreover, the number of observations per cluster was the same in all simulations, making it impossible to draw conclusions about effects of ignoring clustering where cluster sizes vary (including situations where cluster size is informative [24]). Also, data were simulated following the specification of the RI regression model (as described in Eq. 2) rather than that of the random-effects model. That was done because the RI model is more frequently used, especially when there is no prior expectation of differential effects of the explanatory on the outcome variable across different clusters. Simulating data following the specification of the random effects model would have added to the complexity of the algorithm used for simulation, and to the computational time required.

Given the method by which the simulated samples were generated, it was to be expected that when multilevel RI modelling was applied, irrespective of whether the explanatory variable was continuous or binary, the rate of Type I error would be 5%, and the coverage by 95% CIs would be at the nominal level of 95%. In comparison, when cluster-unadjusted OLS models were fitted to clustered data with a continuous ${x}_{ij}$, rates of Type I error were higher, particularly when the ICC was high. For the highest level of ICC examined (0.3), Type I errors were as frequent as 70%. However, even with an ICC of only 0.01, rates of Type I error were more than 10%. Consistent with this, coverage by 95% confidence intervals was much lower than the nominal value (rates down to 30%) when ICC levels were high. In contrast to these results Huang et al. [25] have reported coverage close to 95% from the OLS model when it was applied to clustered data with a continuous explanatory variable. Differences between our findings and those of Huang et al. [25] may be explained by lack of clustering in the explanatory variable in Huang’s investigation. Sensitivity analysis restricting our simulated datasets to those in which clustering of explanatory variable was minimal showed that interval coverage rates were close to 95%, independent of clustering in the outcome variable (Supplementary Table 4).

When ${x}_{ij}$ was binary and OLS regression was applied, interval coverage and rates of Type I error varied little around the nominal values of 95 and 5%, and only for ICC values higher than 0.01. Overall coverage rates were above the nominal rate for higher ICCs and decreased with greater dispersion of the prevalence of ${x}_{ij}$ across clusters, and with lower overall prevalence of the ${x}_{ij}$. A similar observation of small variation of interval coverage around 95% for higher ICC values has been reported previously [26]. Type I error when ${x}_{ij}$ was binary and its overall prevalence low, varied around 5% with values below 5% for small dispersion of prevalence of ${x}_{ij}$ across clusters, and above 5% for large dispersion. For high overall prevalence of ${x}_{ij}$ (up to 0.4), Type I error rates fell below 5%. In accordance with these findings, Galbraith et al. [27] have shown that cluster-unadjusted models resulted in relatively conservative Type I error. Also, in a context of individually randomised trials, Kahan et al. [28] have shown that Type I error increased with increasing ICC and increasing difference in the probability of assignment of patients to treatment arms.

It has been widely stated that when data are clustered, effects estimated by OLS regression are unbiased [22, 26, 29–31], at least where cluster size is uninformative, and there is no confounding by cluster [32]. Our results confirm that for data of the type simulated (in which the clusters were all of equal size and effect sizes did not vary by cluster), coefficients from both OLS and RI regression were on average very similar to the “true” value that had been used in generating simulated samples. Previous studies based on simulated data have shown similar results [22, 25, 26, 33]. However, for individual simulated samples, effect estimates often differed from the “true” value, with larger deviations for a continuous explanatory variable when the OLS model was fitted. For continuous ${x}_{ij}$, the potential magnitude of deviations from the “true” value depended on the extent to which the outcome variable was clustered. For an ICC of 0.3, OLS estimates of effect differed by up to 10% from the “true” value (Fig. 2A). In addition, when ${x}_{ij}$ was continuous, the error in OLS estimates of the regression coefficient was largest when the between-cluster dispersion of ${x}_{ij}$ was similar to that within-clusters (the within-cluster SD of ${x}_{ij}$ having been arbitrarily set to 1 in the simulation algorithm). When ${x}_{ij}$ was binary, the deviation of OLS estimates from the “true” value increased as the dispersion of prevalence rates across clusters increased, and when the target prevalence rate across all clusters was lower (< 10%) (Supplementary Table 2). These errors in effect estimates indicate that in an individual study, failure of regression analysis to account for clustering of observations could result in substantially higher or lower estimates of effect than those derived from multilevel analysis. This has been illustrated in numerous published papers of real data, which have shown that estimates from the two analytical methods can differ to a lesser or greater extent [10, 15, 18, 20, 34]. However, in those publications, little or no information was provided to establish how the observed error related to clustering of the explanatory variable.

The ratio of the SE of a regression coefficient estimated from the RI model to that derived when an OLS model is applied to the same sample, provides an inverse measure of their relative precision. Given that effects estimated from both RI and OLS models were unbiased, and that coverage of confidence intervals derived from RI models was consistently close to the nominal value of 95%, deviation of the measure from unity is likely to reflect bias in SEs estimated by OLS regression. It is widely stated that regression coefficients are spuriously precise when clustering is not taken into account in regression models, although authors have often failed to specify the conditions under which this applies [20, 33, 35–38]. Other authors have pointed out that when ${x}_{ij}$ is identical within each cluster (as, for example, in a cluster-randomised trial), and a cluster-unadjusted approach is followed, SEs tend to be spuriously low, and that the opposite occurs when ${x}_{ij}$ varies within clusters [25, 28, 39, 40]. Higher SEs from OLS models for effects of explanatory variables that varied within clusters have been demonstrated in analyses of both real and simulated data [34, 41]. Others, however, have reported contradictory results in which SEs of effect estimates from OLS regression where ${x}_{ij}$ varied within clusters were very similar to, or lower than, those from a multi-level model [15–18, 42]. It should be noted that the dichotomy between cluster- and individual-level variables is not clear-cut. There can be varying degrees of clustering in ${x}_{ij}$, the extremes occurring where its mean value is the same for all clusters (i.e. it is completely unclustered), and where it does not vary at all within clusters (i.e. it is a cluster-specific or cluster-constant variable). However, in observational studies, an explanatory variable can lie anywhere between these extremes. In recognition of this, an early paper focused on the level of clustering in ${x}_{ij}$ as a driver for the expected bias in the precision of effect estimates [29], rather than making a dichotomous distinction between cluster-constant and cluster-varying ${x}_{ij}$. The authors reported that as clustering in ${x}_{ij}$ decreases, the bias in SEs from a cluster-unadjusted model is expected to increase, and vice versa. Taking into consideration clustering in ${x}_{ij}$ as well as in the outcome variable, a later study using simulated data showed that for a given level of clustering in the outcome variable, increasing the clustering of the explanatory variable caused the ratio of estimated SEs (${SE}_{\beta }^{RI}/{SE}_{\beta }^{OLS}$) to increase from values < 1 to values $\approx 1$ [43]. Our results for continuous explanatory variables differ slightly from this, with ratios of SEs (${SE}_{\beta }^{RI}/{SE}_{\beta }^{OLS}$) moving from values < 1 to values > 1, as clustering of the explanatory variable, expressed as dispersion of ${\stackrel{-}{x}}_{j}$ across clusters, increased. This accords with the finding that OLS regression will have spuriously high precision when it is used to analyse cluster-randomised trials, and spuriously low precision when it is applied to data from individually randomised trials [28, 39, 40].

Bias in the precision of effect estimates for binary ${x}_{ij}$ when clustering is ignored has received only limited attention in the published literature. Several reported studies have compared standard and multi-level models, using real data with continuous and binary ${x}_{ij}$ that varied within clusters [15, 18]. Where ${x}_{ij}$ was binary, SEs derived from the OLS model were mostly larger than those from the multi-level model. The same conclusion was drawn from a study using simulated data [26]. However, neither of the studies using real data has explored the level of bias in relation to variation in the prevalence of the binary ${x}_{ij}$, and the study of simulated data assumed constant prevalence of ${x}_{ij}$ in all clusters.

In our analyses, SEs from the multi-level model were generally lower than those from the OLS model, irrespective of the dispersion of prevalence of ${x}_{ij}$ across clusters and its overall prevalence. This contrasted with findings when ${x}_{ij}$ was continuous, where ratios of SEs (${SE}_{\beta }^{RI}/{SE}_{\beta }^{OLS}$) increased from < 1 to > 1, as the dispersion of ${\stackrel{-}{x}}_{j}$ across clusters increased. The explanation for the discrepancy may lie in the ranges of dispersion of ${\stackrel{-}{x}}_{j}$ across clusters that were explored for continuous as compared with binary ${x}_{ij}$. For a continuous explanatory variable ${x}_{ij}$, we allowed wide dispersion of ${\stackrel{-}{x}}_{j}$ across clusters, while for binary ${x}_{ij}$ the dispersion of ${\stackrel{-}{x}}_{j}$ across clusters was constrained to low levels in order to achieve overall prevalence rates for ${x}_{ij}$ that were close to the target values (this applied particularly where the target prevalence was 5%). The ratio of SEs (${SE}_{\beta }^{RI}/{SE}_{\beta }^{OLS}$) for continuous ${x}_{ij}$ only clearly exceeded one for dispersions of ${\stackrel{-}{x}}_{j}$ across clusters greater than those that were examined for binary ${x}_{ij}$.

The focus of this paper was on the association between a continuous outcome and an explanatory variable ${x}_{ij}$ that varied within clusters. We showed that when ${x}_{ij}$ was continuous, and most of its variation was within rather than between clusters, the cluster-unadjusted OLS model gave larger SEs for the regression coefficient than multi-level modelling. This accords with reports that ignoring clustering can lead to spuriously high SEs when ${x}_{ij}$ varies within clusters. The reverse occurred when most of the dispersion of ${x}_{ij}$ was between rather than within clusters, a situation approaching that of a cluster-specific explanatory variable. We additionally showed that when ${x}_{ij}$ was binary, ignoring clustering in statistical modelling in most cases resulted in higher SEs for the estimated effect than those derived from the random-intercept model, possibly reflecting low simulated dispersion of the prevalence of ${x}_{ij}$ across clusters. The SEs differed more for higher ICCs but not with the overall prevalence of ${x}_{ij}$, nor with the dispersion of its prevalence across clusters (Fig. 3B). Unlike SEs, the point estimates were unbiased for both continuous or binary ${x}_{ij}$ (Fig. 2A and B).

Thus, our results support the use of multi-level modelling to account for clustering effects in linear regression analyses of data that are hierarchically structured with characteristics similar to those that we explored (large sample size, similarly sized clusters, and no variation in the effects of explanatory variables by cluster), especially where ICCs might exceed 0.01. Failure to do so is likely to result in incorrect estimates of effect (either too high or too low) with spuriously high or low precision according to the level of clustering of explanatory variables, and thus may lead to incorrect inferences. The errors in estimates of effect of a continuous ${x}_{ij}$ will be smaller when most of its dispersion is between rather than within clusters – i.e. the variable comes closer to being cluster-specific. When ${x}_{ij}$ is binary, smaller errors in the effect estimates occur when its overall prevalence $p$ across clusters is closer to 50%, i.e. when the variance of the binary variable is at its maximum ($p\left(1-p\right)=25\%$).

Additionally, we have identified circumstances in which a simpler analytical approach that does not adjust for clustering is more likely to mislead statistical inference, i.e. in which rates of Type I error and interval coverage deviate materially from the nominal values of 5 and 95% respectively. These occur when ${x}_{ij}$ is continuous, and ICC levels are greater than 0.01. It is then that Type I error rates are higher than 10% and interval coverage rates are lower than 80%. Statistical inference when a standard regression model is fitted is less likely to be problematic when ${x}_{ij}$ is binary, but again Type I error rates can sometimes be greater than 10%, and corresponding interval coverage rates lower than 90%. This occurs when ICC is high, the overall prevalence of ${x}_{ij}$ is low (approximately 5%), and the dispersion of the cluster-specific prevalence of ${x}_{ij}$ is large. In all circumstances in which the ICC is small, clustering is minimal and there is little difference between RI and OLS regression.

## Supplementary Information

**Additional file 1: Appendix A.** Data generating algorithm for clustered data with continuous outcome and explanatory variables. **Appendix B.** Data generating algorithm for clustered data with continuous outcome and binary explanatory variables. **Supplementary Table 1.** Standard deviation of derived ${\beta }_{1}^{RI}$ and ${\beta }_{1}^{OLS}$ when ${x}_{ij}$ was continuous according to fifths of the distribution of dispersion (expressed as SD) of the continuous ${\stackrel{-}{x}}_{j}$. **Supplementary Table 2.** Standard deviation of derived ${\beta }_{1}^{RI}$ and ${\beta }_{1}^{OLS}$ when ${x}_{ij}$ was binary according to fifths of the distribution of dispersion (expressed as SD) of the prevalence of ${x}_{j}$ across clusters, and overall target prevalence of ${x}_{ij}$. **Supplementary Table 3.** Percentage (%) of datasets for which the null hypothesis was rejected according to level of ICC when ${\beta }_{1}=0$ and ${x}_{ij}$ was continuous according to fifths of the distribution of dispersion (expressed as SD) of the continuous ${\stackrel{-}{x}}_{j}$. **Supplementary Table 4.** Interval coverage rates by ICC levels when clustering in the continuous explanatory variable was minimal (dispersion of the continuous ${\stackrel{-}{x}}_{j}$<0.2 SDs).

## Data Availability

The simulated datasets used and analysis described in the current study are available from the corresponding author on reasonable request.

## Abbreviations

RI: Random-intercept
OLS: Ordinary least squares
ICC: Intra-class correlation coefficient
SE: Standard error

## References

[1] Rabe-Hesketh S, Skrondal A. Multilevel and longitudinal modeling using stata. USA: Taylor & Francis; 2005.

[2] Stimson JA (1985). Regression in space and time: a statistical essay. Am J Pol Sci.

[3] Bingenheimer JB, Raudenbush SW (2004). Statistical and substantive inferences in public health: issues in the application of multilevel models. Annu Rev Public Health.

[4] Goldstein H. Multilevel statistical models. United Kingdom: Wiley; 2011.

[5] McNeish D, Kelley K (2019). Fixed effects models versus mixed effects models for clustered data: reviewing the approaches, disentangling the differences, and making recommendations. Psychol Methods.

[6] Bland JM (2004). Cluster randomised trials in the medical literature: two bibliometric surveys. BMC Med Res Methodol.

[7] Crits-Christoph P, Mintz J (1991). Implications of therapist effects for the design and analysis of comparative studies of psychotherapies. J Consult Clin Psychol.

[8] Lee KJ, Thompson SG (2005). Clustering by health professional in individually randomised trials. BMJ (Clinical research ed).

[9] Simpson JM, Klar N, Donnor A (1995). Accounting for cluster randomization: a review of primary prevention trials, 1990 through 1993. Am J Public Health.

[10] Biau DJ, Halm JA, Ahmadieh H, Capello WN, Jeekel J, Boutron I (2008). Provider and center effect in multicenter randomized controlled trials of surgical specialties: an analysis on patient-level data. Ann Surg.

[11] Oltean H, Gagnier JJ (2015). Use of clustering analysis in randomized controlled trials in orthopaedic surgery. BMC Med Res Methodol.

[12] Diaz-Ordaz K, Froud R, Sheehan B, Eldridge S (2013). A systematic review of cluster randomised trials in residential facilities for older people suggests how to improve quality. BMC Med Res Methodol.

[13] Goldstein H (1986). Multilevel mixed linear model analysis using iterative generalized least squares. Biometrika.

[14] Astin AW, Denson N (2009). Multi-campus studies of college impact: which statistical method is appropriate?. Res High Educ.

[15] Grieve R, Nixon R, Thompson SG, Normand C (2005). Using multilevel models for assessing the variability of multinational resource use and cost data. Health Econ.

[16] Niehaus E, Campbell C, Inkelas K (2014). HLM behind the curtain: unveiling decisions behind the use and interpretation of HLM in higher education research. Res High Educ.

[17] Steenbergen MR, Jones BS. Modeling multilevel data structures. Am J Pol Sci. 2002;46(1):218–37.

[18] Wendel-Vos GCW, van Hooijdonk C, Uitenbroek D, Agyemang C, Lindeman EM, Droomers M (2008). Environmental attributes related to walking and bicycling at the individual and contextual level. J Epidemiol Community Health.

[19] Walters SJ (2010). Therapist effects in randomised controlled trials: what to do about them. J Clin Nurs.

[20] Park S, Lake ET (2005). Multilevel modeling of a clustered continuous outcome: nurses’ work hours and burnout. Nurs Res.

[21] Newman D, Newman I, Salzman J (2010). Comparing OLS and HLM models and the questions they answer: potential concerns for type VI errors. Mult Linear Regression Viewpoints.

[22] Clarke P (2008). When can group level clustering be ignored? Multilevel models versus single-level models with sparse data. J Epidemiol Community Health.

[23] Bradburn MJ, Deeks JJ, Berlin JA, Russell LA (2007). Much ado about nothing: a comparison of the performance of meta-analytical methods with rare events. Stat Med.

[24] Nevalainen J, Datta S, Oja H (2014). Inference on the marginal distribution of clustered data with informative cluster size. Stat Pap.

[25] Huang FL (2016). Alternatives to multilevel modeling for the analysis of clustered data. J Exp Educ.

[26] Chu R, Thabane L, Ma J, Holbrook A, Pullenayegum E, Devereaux PJ (2011). Comparing methods to estimate treatment effects on a continuous outcome in multicentre randomized controlled trials: a simulation study. BMC Med Res Methodol.

[27] Galbraith S, Daniel J, Vissel B (2010). A study of clustered data and approaches to its analysis. J Neurosci.

[28] Kahan BC, Morris TP (2013). Assessing potential sources of clustering in individually randomised trials. BMC Med Res Methodol.

[29] Arceneaux K, Nickerson DW (2009). Modeling certainty with clustered data: a comparison of methods. Polit Anal.

[30] Scott AJ, Holt D (1982). The effect of two-stage sampling on ordinary least squares methods. J Am Stat Assoc.

[31] Barrios T, Diamond R, Imbens GW, Koleśar M (2012). Clustering, spatial correlations, and randomization inference. J Am Stat Assoc.

[32] Seaman S, Pavlou M, Copas A (2014). Review of methods for handling confounding by cluster and informative cluster size in clustered data. Stat Med.

[33] Maas CJ, Hox JJ (2004). The influence of violations of assumptions on multilevel parameter estimates and their standard errors. Comput Stat Data Anal.

[34] Dickinson LM, Basu A (2005). Multilevel modeling and practice-based research. Ann Fam Med.

[35] Austin PC, Goel V, van Walraven C (2001). An introduction to multilevel regression models. Can J Public Health.

[36] Lemeshow S, Letenneur L, Dartigues JF, Lafont S, Orgogozo JM, Commenges D (1998). Illustration of analysis taking into account complex survey considerations: the association between wine consumption and dementia in the PAQUID study. Am J Epidemiol.

[37] Roberts C, Roberts SA (2005). Design and analysis of clinical trials with clustering effects due to treatment. Clin Trials.

[38] Maas CJ, Hox JJ (2005). Sufficient sample sizes for multilevel modeling. Methodology.

[39] Chuang JH, Hripcsak G, Heitjan DF (2002). Design and analysis of controlled trials in naturally clustered environments: implications for medical informatics. JAMIA.

[40] Sainani K (2010). The importance of accounting for correlated observations. PM&R.

[41] Jones K (2009). Do multilevel models ever give different results?.

[42] Hedeker D, McMahon SD, Jason LA, Salina D (1994). Analysis of clustered data in community psychology: with an example from a worksite smoking cessation project. Am J Community Psychol.

[43] Bliese PD, Hanges PJ (2004). Being both too liberal and too conservative: the perils of treating grouped data as though they were independent. Organ Res Methods.

