# Supplementary material for: Consequences of ignoring clustering in linear regression
Source: BMC Med Res Methodol. 2021 Jul 7;21:139. doi: 10.1186/s12874-021-01333-7 (PMC8265092; doi:10.1186/s12874-021-01333-7)
Supplement: Supplementary file 1 — Additional file 1: Appendix A. Data generating algorithm for clustered data with continuous outcome and explanatory variables. Appendix B. Data generating algorithm for clustered data with continuous outcome and binary explanatory variables. Supplementary Table 1. Standard deviation of derived ${\beta }_{1}^{RI}$β1RI and ${\beta }_{1}^{OLS}$β1OLS when ${x}_{ij}$xij was continuous according to fifths of the distribution of dispersion (expressed as SD) of the continuous ${\stackrel{-}{x}}_{j}$x-j. Supplementary Table 2. Standard deviation of derived ${\beta }_{1}^{RI}$β1RI and [file 12874_2021_1333_MOESM1_ESM.docx]

**Supplementary material**

**Appendix A.** Data generating algorithm for clustered data with continuous outcome and explanatory variables

**Appendix B.** Data generating algorithm for clustered data with continuous outcome and binary explanatory variables

**Supplementary Table 1.** Standard deviation of derived $\beta_{1}^{RI}$ and $\beta_{1}^{OLS}$ when $x_{ij}$ was continuous according to fifths of the distribution of dispersion (expressed as SD) of the continuous $\bar{x}_{j}$

**Supplementary Table 2.** Standard deviation of derived $\beta_{1}^{RI}$ and $\beta_{1}^{OLS}$ when $x_{ij}$ was binary according to fifths of the distribution of dispersion (expressed as SD) of the prevalence of $x_{j}$ across clusters, and overall target prevalence of $x_{ij}$

**Supplementary Table 3.** Percentage (%) of datasets for which the null hypothesis was rejected according to level of ICC when $\beta_{1}=0$ and $x_{ij}$ was continuous according to fifths of the distribution of dispersion (expressed as SD) of the continuous $\bar{x}_{j}$

**Supplementary Table 4.** Interval coverage rates by ICC levels when clustering in the continuous explanatory variable was minimal (dispersion of the continuous $\bar{x}_{j}$<0.2 SDs)

**Appendix A**

**Data generating algorithm for clustered data with continuous outcome and explanatory variables**

Step 1: Generate vector of 1000 values $K\sim U\left[ 0,20 \right]$

Step 2: Set $sd_{shift}$ to the first value of vector $K$, and ${sd}_{e}=1$

Step 3: Set up hierarchical data of 100 clusters (clusters are indicated as $j$, with $j=1,\ldots,100$) with 100 observations per cluster (observations within each cluster are indicated as $i$, with $i=1,\ldots,100$ per $j$ value)

Step4: Generate the following variables

1. variable $u_{j}$ that varies only across clusters (cluster-specific error term), such that $u_{j}\sim N\left( 0, {sd_{u}}^{2} \right)$, with $sd_{u}=0.0316$ for a target ICC within the range (0.0005-0.00149) assuming ${sd}_{e}=1$ (as per Step 2)
2. variable ${shift}_{j}$ that varies only across clusters, such that ${shift}_{j}\sim N\left( 0, {{sd}_{shift}}^{2} \right)$
3. variable $e_{ij}$ that varies across and within clusters, such that $e_{ij}\sim N\left( 0, 1 \right)$ and $corr\left( e_{ij},u_{j} \right)=0$
4. independent variable $x_{0ij}$, such that $x_{0ij}\sim N\left( 0, 1 \right)$
5. independent variable $x_{ij}=x_{0ij}+{shift}_{j}$
6. dependent variable $y_{ij}$ as $y_{ij}=ϐ_{0}+ϐ_{1}x_{ij}+e_{ij}+u_{j}=x_{ij}+e_{ij}+u_{j}$ with $ϐ_{0}=0$ and $ϐ_{1}=1$

Step 5: Fit the linear random-intercept (RI) model and estimate ICC.

Step 6: If ICC is by chance outside the pre-defined target range of ICC (as defined in Step 4i), drop simulated dataset and restart from Step 3

Else

Fit the RI and OLS models and save effect estimates and their corresponding standard errors, and estimated ICC

Step 7: Drop all variables generated in Step 4, and repeat Step 4 – Step 6 to generate 100 datasets for given $sd_{u}$ (and thus for given ICC within the range specified in Step 4 i) and $sd_{shift}$ (specified in Step 2)

Step 8: Repeat steps 2-7 for the 1000 values of $sd_{shift}$ (derived in Step 1)

Step 9: Repeat steps 1-8 for $sd_{u}=0.0549$ for a target ICC within the range (0.0025-0.00349)

Step 10: Repeat steps 1-8 for $sd_{u}=0.1005$for a target ICC within the range (0.005-0.0149)

Step 11: Repeat steps 1-8 for $sd_{u}=0.1759$ for a target ICC within the range (0.025-0.0349)

Step 12: Repeat steps 1-8 for $sd_{u}=0.3333$for a target ICC within the range (0.05-0.149)

Step 13: Repeat steps 1-8 for $sd_{u}=0.6547$for a target ICC within the range (0.25-0.349)

Step 14: Repeat steps 1-13 with $ϐ_{1}=0$ in Step 4vi

**Appendix B**

**Data generating algorithm for clustered data with continuous outcome and binary explanatory variables**

Step 1: Set overall prevalence of the explanatory variable$prev_{x0}=0.05$

Step 2: Generate vector of 500 values $K\sim U\left[ 0,0.05 \right]$

Step 3: Set $sd_{shift}$ to the first value of vector $K$, and ${sd}_{e}=1$

Step 4: Set up hierarchical data of 100 clusters (clusters are indicated as $j$, with $j=1,\ldots,100$) with 100 observations per cluster (observations within each cluster are indicated as $i$, with $i=1,\ldots,100$ per $j$ value)

Step 5: Generate the following variables

1. variable $u_{j}$ that varies only across clusters (cluster-specific error term), such that $u_{j}\sim N\left( 0, {sd_{u}}^{2} \right)$, with $sd_{u}=0.0316$ for a target ICC within the range (0.0005-0.00149) assuming ${sd}_{e}=1$ (as per Step 3)
2. variable ${shift}_{j}$ that varies only across clusters, such that ${shift}_{j}\sim N\left( 0, {{sd}_{shift}}^{2} \right)$
3. variable $e_{ij}$ that varies across and within clusters, such that $e_{ij}\sim N\left( 0, 1 \right)$ and $corr\left( e_{ij},u_{j} \right)=0$
4. cluster prevalence of the explanatory variable $prev_{x_{j}}=prev_{x0}+{shift}_{j}$, where $prev_{x0}=0.05$ as defined in Step 1. If $prev_{x_{j}}<0$, reset to 0. Then, within each cluster, set $x_{ij}$ to 1 for the first $n$ observations, with $n<mean(prev_{x_{j}})$, and 0 otherwise
5. dependent variable $y_{ij}$ as $y_{ij}=ϐ_{0}+ϐ_{1}x_{ij}+e_{ij}+u_{j}=x_{ij}+e_{ij}+u_{j}$ with $ϐ_{0}=0$ and $ϐ_{1}=1$

Step 6: Fit the linear random-intercept (RI) model and estimate ICC.

Step 7: If ICC is by chance outside the pre-defined target range of ICC (as defined in Step 5 i), drop simulated dataset and restart from Step 4

Else

Fit the RI and OLS models and save effect estimates, their corresponding standard errors, and estimated ICC

Step 8: Drop all variables generated in Step 5, and repeat Step 5 – Step 7 to gen 100 datasets for given $sd\_u$ (and thus for given ICC within the range specified in Step 5 i) and $sd_{shift}$ (specified in Step 3)

Step 9: Repeat steps 2-8 for the 500 values of $sd_{shift}$ (derived in Step 2)

Step 10: Repeat steps 2-9 for $sd_{u}=0.0549$ for a target ICC within the range (0.0025-0.00349)

Step 11: Repeat steps 2-9 for $sd_{u}=0.1005$for a target ICC within the range (0.005-0.0149)

Step 12: Repeat steps 2-9 for $sd_{u}=0.1759$ for a target ICC within the range (0.025-0.0349)

Step 13: Repeat steps 2-9 for $sd_{u}=0.3333$for a target ICC within the range (0.05-0.149)

Step 14: Repeat steps 2-9 for $sd_{u}=0.6547$for a target ICC within the range (0.25-0.349)

Step 15: Repeat steps 1-14 for $prev_{x0}=0.1$

Step 16: Repeat steps 1-14 for $prev_{x0}=0.2$

Step 17: Repeat steps 1-14 for $prev_{x0}=0.4$

Step18: Repeat steps 1-15 with $ϐ_{1}=0$ in Step 5v

**Supplementary Table 1.** Standard deviation of derived $\beta_{1}^{RI}$ and $\beta_{1}^{OLS}$ when $x_{ij}$ was continuous according to fifths of the distribution of dispersion (expressed as SD) of the continuous $\bar{x}_{j}$

| **ICC** | **1^st^** | | **2^nd^** | | **3^rd^** | | **4^th^** | | **5^th^** | |
| --- | --- | --- | --- | --- | --- | --- | --- | --- | --- | --- |
|  | **RI** | **OLS** | **RI** | **OLS** | **RI** | **OLS** | **RI** | **OLS** | **RI** | **OLS** |
| **0.001** | 0.007 | 0.007 | 0.002 | 0.002 | 0.001 | 0.001 | 0.001 | 0.001 | 0.001 | 0.001 |
| **0.003** | 0.007 | 0.007 | 0.003 | 0.003 | 0.002 | 0.002 | 0.001 | 0.001 | 0.001 | 0.001 |
| **0.01** | 0.007 | 0.008 | 0.003 | 0.003 | 0.002 | 0.002 | 0.001 | 0.001 | 0.001 | 0.001 |
| **0.03** | 0.008 | 0.010 | 0.004 | 0.005 | 0.003 | 0.003 | 0.002 | 0.002 | 0.002 | 0.002 |
| **0.1** | 0.009 | 0.015 | 0.006 | 0.008 | 0.004 | 0.005 | 0.003 | 0.003 | 0.003 | 0.003 |
| **0.3** | 0.010 | 0.028 | 0.009 | 0.015 | 0.007 | 0.009 | 0.006 | 0.007 | 0.005 | 0.005 |

**Supplementary Table 2.** Standard deviation of derived $\beta_{1}^{RI}$ and $\beta_{1}^{OLS}$ when $x_{ij}$ was binary according to fifths of the distribution of dispersion (expressed as SD) of the prevalence of $x_{j}$ across clusters, and overall target prevalence of $x_{ij}$

|  | **ICC** | **1^st^** | | **2^nd^** | | **3^rd^** | | **4^th^** | | **5^th^** | |
| --- | --- | --- | --- | --- | --- | --- | --- | --- | --- | --- | --- |
|  |  | **RI** | **OLS** | **RI** | **OLS** | **RI** | **OLS** | **RI** | **OLS** | **RI** | **OLS** |
| **Prevalence of x = 0.05** | **0.001** | 0.046 | 0.046 | 0.046 | 0.046 | 0.046 | 0.046 | 0.045 | 0.045 | 0.044 | 0.044 |
|  | **0.003** | 0.046 | 0.046 | 0.046 | 0.046 | 0.045 | 0.045 | 0.046 | 0.046 | 0.045 | 0.045 |
|  | **0.01** | 0.046 | 0.046 | 0.045 | 0.045 | 0.046 | 0.046 | 0.046 | 0.046 | 0.045 | 0.045 |
|  | **0.03** | 0.046 | 0.046 | 0.046 | 0.046 | 0.046 | 0.047 | 0.046 | 0.047 | 0.045 | 0.046 |
|  | **0.1** | 0.046 | 0.046 | 0.045 | 0.047 | 0.046 | 0.049 | 0.046 | 0.051 | 0.045 | 0.052 |
|  | **0.3** | 0.046 | 0.047 | 0.046 | 0.050 | 0.046 | 0.057 | 0.046 | 0.065 | 0.046 | 0.071 |
| **Prevalence of x = 0.1** | **0.001** | 0.033 | 0.033 | 0.033 | 0.033 | 0.033 | 0.033 | 0.034 | 0.034 | 0.033 | 0.033 |
|  | **0.003** | 0.033 | 0.033 | 0.034 | 0.034 | 0.033 | 0.033 | 0.034 | 0.034 | 0.033 | 0.033 |
|  | **0.01** | 0.033 | 0.033 | 0.033 | 0.033 | 0.034 | 0.034 | 0.034 | 0.034 | 0.033 | 0.034 |
|  | **0.03** | 0.033 | 0.033 | 0.033 | 0.033 | 0.034 | 0.034 | 0.034 | 0.034 | 0.034 | 0.034 |
|  | **0.1** | 0.033 | 0.033 | 0.034 | 0.034 | 0.033 | 0.035 | 0.034 | 0.036 | 0.034 | 0.037 |
|  | **0.3** | 0.034 | 0.034 | 0.034 | 0.036 | 0.033 | 0.038 | 00.034 | 0.046 | 0.034 | 0.046 |
| **Prevalence of x = 0.2** | **0.001** | 0.025 | 0.025 | 0.025 | 0.025 | 0.025 | 0.025 | 0.025 | 0.025 | 0.025 | 0.025 |
|  | **0.003** | 0.025 | 0.025 | 0.025 | 0.025 | 0.025 | 0.025 | 0.025 | 0.025 | 0.025 | 0.025 |
|  | **0.01** | 0.025 | 0.025 | 0.025 | 0.025 | 0.025 | 0.025 | 0.025 | 0.025 | 0.025 | 0.025 |
|  | **0.03** | 0.025 | 0.025 | 0.025 | 0.025 | 0.025 | 0.025 | 0.025 | 0.025 | 0.025 | 0.026 |
|  | **0.1** | 0.025 | 0.025 | 0.025 | 0.025 | 0.025 | 0.025 | 0.025 | 0.026 | 0.025 | 0.027 |
|  | **0.3** | 0.025 | 0.025 | 0.025 | 0.026 | 0.025 | 0.027 | 0.025 | 0.029 | 0.025 | 0.031 |
| **Prevalence of x = 0.4** | **0.001** | 0.020 | 0.020 | 0.020 | 0.020 | 0.021 | 0.021 | 0.021 | 0.021 | 0.020 | 0.020 |
|  | **0.003** | 0.020 | 0.020 | 0.020 | 0.020 | 0.020 | 0.020 | 0.020 | 0.020 | 0.021 | 0.021 |
|  | **0.01** | 0.020 | 0.020 | 0.020 | 0.020 | 0.021 | 0.021 | 0.021 | 0.021 | 0.020 | 0.020 |
|  | **0.03** | 0.020 | 0.020 | 0.020 | 0.020 | 0.021 | 0.021 | 0.021 | 0.021 | 0.021 | 0.021 |
|  | **0.1** | 0.020 | 0.020 | 0.021 | 0.021 | 0.020 | 0.021 | 0.020 | 0.021 | 0.021 | 0.021 |
|  | **0.3** | 0.020 | 0.021 | 0.021 | 0.021 | 0.020 | 0.021 | 0.020 | 0.022 | 0.021 | 0.024 |

**Supplementary Table 3.** Percentage (%) of datasets for which the null hypothesis was rejected according to level of ICC when $\beta_{1}=0$ and $x_{ij}$ was continuous according to fifths of the distribution of dispersion (expressed as SD) of the continuous $\bar{x}_{j}$

| **ICC** | **1^st^** | | **2^nd^** | | **3^rd^** | | **4^th^** | | **5^th^** | | **Total** | |
| --- | --- | --- | --- | --- | --- | --- | --- | --- | --- | --- | --- | --- |
|  | **RI** | **OLS** | **RI** | **OLS** | **RI** | **OLS** | **RI** | **OLS** | **RI** | **OLS** | **RI** | **OLS** |
| **0.001** | 5.00 | 5.72 | 4.74 | 5.82 | 5.15 | 6.11 | 4.86 | 5.93 | 4.70 | 5.93 | 4.90 | 5.91 |
| **0.003** | 4.81 | 7.04 | 4.53 | 7.79 | 4.80 | 8.04 | 4.92 | 8.25 | 4.75 | 8.09 | 4.76 | 7.85 |
| **0.01** | 5.05 | 12.27 | 5.26 | 16.34 | 5.24 | 16.50 | 5.25 | 16.15 | 5.41 | 16.51 | 5.24 | 15.57 |
| **0.03** | 5.21 | 23.96 | 5.13 | 31.74 | 5.33 | 32.92 | 5.38 | 32.65 | 5.35 | 32.32 | 5.28 | 30.78 |
| **0.1** | 5.29 | 43.94 | 5.01 | 54.94 | 5.18 | 55.51 | 5.37 | 55.31 | 5.09 | 55.18 | 5.19 | 53.08 |
| **0.3** | 5.02 | 62.65 | 5.31 | 72.36 | 5.55 | 72.45 | 5.21 | 72.63 | 5.26 | 72.37 | 5.26 | 70.33 |

**Supplementary Table 4**. Interval coverage rates by ICC levels when clustering in the continuous explanatory variable was minimal (dispersion of the continuous $\bar{x}_{j}$<0.2 SDs)

| ***ICC*** | ***Coverage (%) by 95% CI under the RI model*** | ***Coverage (%) by 95% CI under the OLS model*** |
| --- | --- | --- |
| *0.001* | *94.86* | *94.96* |
| *0.003* | *95.76* | *95.49* |
| *0.01* | *95.10* | *95.10* |
| *0.03* | *94.49* | *93.82* |
| *0.1* | *95.32* | *93.20* |
| *0.3* | *94.68* | *93.63* |
